# β-Caryophyllene Induces Apoptosis and Inhibits Angiogenesis in Colorectal Cancer Models

**DOI:** 10.3390/ijms221910550

**Published:** 2021-09-29

**Authors:** Saad S. Dahham, Yasser Tabana, Muhammad Asif, Marawan Ahmed, Dinesh Babu, Loiy E. Hassan, Mohamed B. Khadeer Ahamed, Doblin Sandai, Khaled Barakat, Arno Siraki, Amin M. S. A. Majid

**Affiliations:** 1 Department of Science, University of Technology and Applied Sciences, Rustaq 10 P.C:329, Oman; 2 Faculty of Pharmacy and Pharmaceutical Sciences, University of Alberta, Edmonton, AB T6G 2E1, Canada; tabana@ualberta.ca (Y.T.); mmahmed@ualberta.ca (M.A.); kbarakat@ualberta.ca (K.B.); siraki@ualberta.ca (A.S.); 3 Department of Pharmacology, Faculty of Pharmacy, The Islamia University of Bahawalpur, Punjab 63100, Pakistan; Drmasif@iub.edu.pk; 4 Department of Botany, Faculty of Science & Technology, Omdurman Islamic University, P.O. Box 382, Omdurman 14415, Sudan; loiy.ahmed23@oiu.edu; 5 EMAN Research and Testing Laboratory, School of Pharmacy, Universiti Sains Malaysia, Gelugor 11800, Penang, Malaysia; khadeer@emanbio.com (M.B.K.A.); ams@nature-ceuticals.com.au (A.M.S.A.M.); 6 Infectomics Cluster, Advanced Medical and Dental Institute, Universiti Sains Malaysia, Bertam, Kepala Batas 13200, Penang, Malaysia; doblin@usm.my; 7 John Curtin School of Medical Research, College of Medicine, Australian National University, Canberra, ACT 2601, Australia

**Keywords:** Beta-Caryophyllene, colorectal cancer, xenograft nude mice, angiogenesis, VEGFR2

## Abstract

Beta-Caryophyllene (BCP), a naturally occurring sesquiterpene abundantly found in cloves, hops, and cannabis, is the active candidate of a relatively new group of vascular-inhibiting compounds that aim to block existing tumor blood vessels. Previously, we have reported the anti-cancer properties of BCP by utilizing a series of in-vitro anti-tumor-related assays using human colorectal carcinoma cells. The present study aimed to investigate the effects of BCP on in-vitro, ex-vivo, and in-vivo models of anti-angiogenic assays and evaluate its anti-cancer activity in xenograft tumor (both ectopic and orthotopic) mice models of human colorectal cancer. Computational structural analysis and an apoptosis antibody array were also performed to understand the molecular players underlying this effect. BCP exhibited strong anti-angiogenic activity by blocking the migration of endothelial cells, tube-like network formation, suppression of vascular endothelial growth factor (VEGF) secretion from human umbilical vein endothelial cells and sprouting of rat aorta microvessels. BCP has a probable binding at Site#0 on the surface of VEGFR2. Moreover, BCP significantly deformed the vascularization architecture compared to the negative control in a chick embryo chorioallantoic membrane assay. BCP showed a remarkable reduction in tumor size and fluorescence molecular tomography signal intensity in all the mice treated with BCP, in a dose-dependent relationship, in ectopic and orthotopic tumor xenograft models, respectively. The histological analysis of the tumor from BCP-treated mice revealed a clear reduction of the density of vascularization. In addition, BCP induced apoptosis through downregulation of HSP60, HTRA, survivin, and XIAP, along with the upregulation of p21 expressions. These results suggest that BCP acts at multiple stages of angiogenesis and could be used as a promising therapeutic candidate to halt the growth of colorectal tumor cells.

## 1. Introduction

Globally, colorectal cancer (CRC) is one among the major malignant tumors having a high rate of incidence and mortality, nearly about two million new cases were diagnosed, and one million deaths were registered in 2018. These figures represent 9.7% of all cancer diagnoses and 8.9% of all deaths due to cancer in both genders [1]. Despite the rapid progress in diagnostic approach and treatment protocols in recent years, no other cancer kills more men and women than CRC after lung and prostate cancer in the case of men and after breast cancer among women [2]. The World Health Organization (WHO) predicts 77% more new CRC cases in the coming years, resulting in 80% more deaths by 2030 [3]. These data reflect that CRC is a severe health problem, requiring further attention to find new anti-cancer agents and gain more insights into better therapeutic results. Medicinal plants and their derivatives have been shown to have promising potential in the fight against human cancer, both in preventing and treating the patients [4]. Natural products can stop or slow down multiple pathways of carcinogenesis [5], for example, angiogenesis and apoptosis, which are the main driving forces in regulating solid tumors’ development. It is now widely accepted that CRC is an angiogenesis-dependent tumor. High vascular density and stimulation of the vascular endothelial growth factor (VEGF) are linked to cancer cell proliferation, migration, local tissue invasion, and distant metastasis, which are the factors threatening the patients’ chances of survival [6]. Moreover, VEGF’s sustained angiogenesis and expression are engaged in the activation of pro-survival regulatory markers (such as the Bcl family proteins and Mcl-1 genes) in various solid tumors. For instance, anti-apoptotic Bcl-2 proteins are directly related to CRC development and therapy resistance [7,8]. With the existence of such an association between angiogenesis and apoptosis, a better understanding of the mechanism(s) underlying colorectal tumorigenesis is a vital step to formulate better cancer therapy. Blocking tumor vascularization by cutting off the blood supply they need and switching off balance in cancer cells through induction of the programmed cell death, both provide a significant and elegant approach in CRC therapy, with the hope of high efficacy and less toxicity [9]. Extensive studies on the central role of the VEGF pathway in angiogenesis have led to the discovery of anti-angiogenic drugs like bevacizumab and cetuximab [6,10], which result in prolonged overall survival of metastatic CRC patients. In the last decade, the advent of immune checkpoint inhibitors is bringing new hopes for treating advanced colonic malignancies [11,12,13].

Beta (β)-caryophyllene (BCP), a naturally occurring sesquiterpene, is abundantly present in cloves, hops, rosemary, numerous essential oils and cannabis. Notably, it is responsible for the spiciness of black pepper, and has been widely used as a safe food flavoring additive with official approval from the Food and Drug Administration [14]. Moreover, BCP is one of the first cannabis-isolated components, which have been shown to selectively bind to the cannabinoid 2 (CB2) receptor [15]. Among the interesting biological activities, BCP was reported to exert a neuroprotective effect in human neuroblastoma SH-SY5Y cells [16]. A rat model study revealed that intraperitoneally injected BCP at the dose of 50 mg/kg for one month has successfully attenuated oxidative stress, inhibited inflammatory mediator cyclooxygenase-2 and reduced neuroinflammation [17]. Furthermore, treatment with BCP has been recently shown to exert a remarkable cardioprotective effect against doxorubicin-induced acute cardiotoxicity in albino Wistar rats [18]. Besides, various in vitro studies have suggested that BCP exerts significant anti-cancer effects against different types of cancer cell lines. In particular, BCP isolated from *Heterotheca inuloides* demonstrated an anti-proliferative effect against the human melanoma (HTB140) and breast cancer (BT-20) cell lines [19]. Another study has reported that BCP is the active compound of *Commiphora gileadensis*, which exhibited an anti-cancer effect against BS-24-1 mouse lymphoma cell line; the activity was related to selective apoptosis induction caused via DNA fragmentation and caspase-3 activation [20]. Furthermore, BCP potentiated the anti-cancer activity of paclitaxel on human colorectal adenocarcinoma (DLD-1), human breast cancer (MCF-7), and murine fibroblast (L-929) cells. Particularly, a combination of BCP has potently increased the anti-cancer properties of paclitaxel against DLD-1 cells by tenfold [21]. Similarly, BCP was reported to potentiate doxorubicin cytotoxicity against the resistant human colorectal adenocarcinoma cells, Caco-2 [22]. Moreover, the synergistic interaction between BCP and some botanical molecules was reported to induce apoptosis of human epidermoid skin cancer cells, mediated by production of reactive oxygen species with loss of mitochondrial membrane potential, as well as alteration of specific apoptotic markers like an increase in Bax/Bcl-2 ratio [23]. We have previously reported that BCP was the active constituent of *Aquilaria crassna*, which displayed a selective anti-cancer activity against the colorectal cancer cell line, HCT 116 [24]. However, the detailed mechanism(s) of how BCP induces apoptosis in CRC need(s) to be elucidated. Besides, whether BCP modulates angiogenesis in vitro and in vivo remains uninvestigated. Therefore, the current study aimed to investigate the anti-tumor activity of BCP against xenograft tumor (ectopic and orthotopic) models of human CRC and the associated mechanism(s) by which it could inhibit the tumorigenesis of CRC cells.

## 2. Results

### 2.1. BCP Inhibited Proliferation and Migration of HUVECs

Our previous study confirmed that BCP was involved in inhibiting the proliferation of colorectal cancer cells, suppressing cancer cell motility, invasion, and colony formation [24]. 

**Figure 1:**
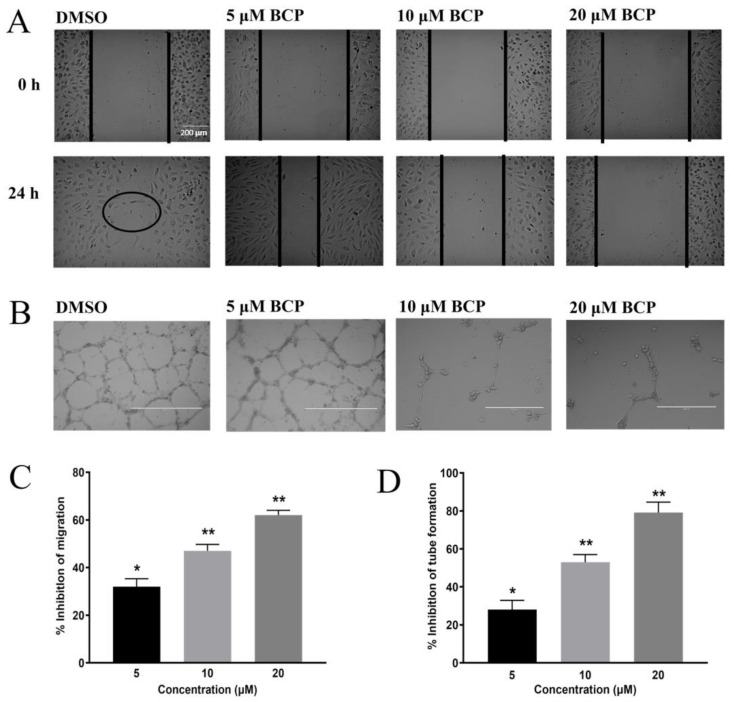
BCP inhibited angiogenesis in vitro. Photomicrographs of scratch assay (**A**) and tube formation assay (**B**) after treatment of 0.5% DMSO, 5, 10, and 20 μM BCP with HUVECs for 24 h. The circle in (**A**) indicates the closure of the wound on the DMSO group at 24 h. The images were captured by an inverted phase-contrast microscope with the scale indicating 200 µm. The concentration-dependent effect of BCP on cell migration assay. Data were represented as mean ± SD (*n* = 3). * *p* < 0.05 and ** *p* < 0.01 compared to DMSO-treated control group (**C**). The concentration-dependent effect of BCP on tube formation assay. Data was represented as mean ± SD (*n* = 3), * *p* < 0.05 and ** *p* < 0.01 compared to DMSO-treated control group (**D**).

In this current study, to investigate the anti-angiogenic activity of BCP in vitro, HUVECs were cultured in 96-well plates and incubated with BCP for 24 h in a concentration range of 6.12 to 100 μM. BCP inhibited the proliferation of HUVECs in a concentration-dependent manner with an IC_50_ of 41.6 ± 3.2 μM. The migration of vascular endothelial cells represents a crucial step toward the development of new blood vessels, allowing the cells to propagate from pre-existing locations and form a network of new vessels. Therefore, the effect of BCP on the motility of HUVECs was investigated by evaluating its effect on wound closure of a scratch wounding model on the endothelial monolayer. Cells on the edge of the newly created wound will move towards the gap to fill the distance until new intercellular contacts are established again. After 24 h, the wound was nearly closed in the cells treated with 0.5% DMSO (as observed by a circle in Figure 1A of 24-h treated group), whereas the wound was still observable in BCP-treated cells. BCP significantly inhibited the motility of HUVECs at 5, 10, and 20 μM with 32.00 ± 3.30%, 47.03 ± 2.72%, and 62.02 ± 2.23% inhibition at 24 h treatment, respectively (Figure 1A,C).

### 2.2. BCP Inhibited Capillary-Like Tube Formation from HUVECs

The anti-angiogenic activity of BCP was evaluated using the tube formation assay, a three-dimensional model of in-vitro angiogenesis. HUVECs were cultured and grown on Matrigel matrix-formed capillary-like tube structure networks. Treatment of HUVECs with BCP significantly attenuated the integrity of the endothelial tube network in a concentration-dependent manner (Figure 1B,D). At a lower concentration of 5 µM, BCP reduced the cell-distance between the capillary-like tubular structures, but partial and fragmented tubules were still observed with an inhibitory effect of 28.03% ± 4.87 (Figure 1B). However, at 10 and 20 μM, BCP significantly abrogated the formation of endothelial tube-like network with an inhibitory effect of 53.06% ± 3.92 and 79.09% ± 5.54, respectively (Figure 1D).

### 2.3. BCP Inhibited VEGF Secretion from HUVECs

**Figure 2:**
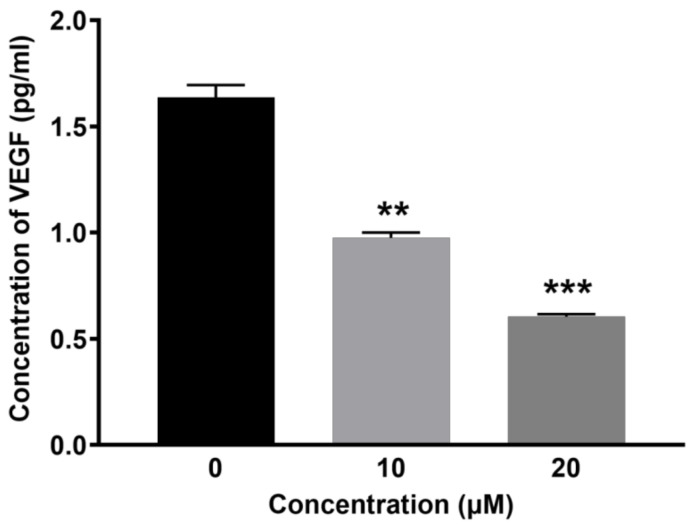
BCP inhibited VEGF secretion in vitro. Graphical representation of the levels of VEGF (pg/mL) after treatment of (0.5% DMSO), 10 and 20 µM BCP with HUVECs for 6 h. Data was represented as mean ± SD (*n* = 3), ** *p* < 0.01 and *** *p* < 0.001 compared to DMSO-treated control group.

Given the observed reduction of endothelial cell motility and tube formation by BCP, the inhibition activity of BCP on VEGF secretion from HUVECs was subsequently measured by quantifying VEGF levels in endothelial cell lysates. BCP significantly inhibited the VEGF levels in HUVECs (Figure 2). The concentration of VEGF in 0.5% DMSO-treated cell lysates (control) was 1.69 ± 1.05 pg/mL, whereas it was lowered in BCP-treated cell lysates to 0.97 ± 1.06 pg/mL and 0.60 ± 1.03 pg/mL at concentrations of 10 and 20 μM of BCP, respectively. Overall, the results clearly suggest that BCP regulates VEGF-mediated angiogenesis in vitro.

### 2.4. BCP Has a Binding Site on the Surface of VEGFR2

**Figure 3:**
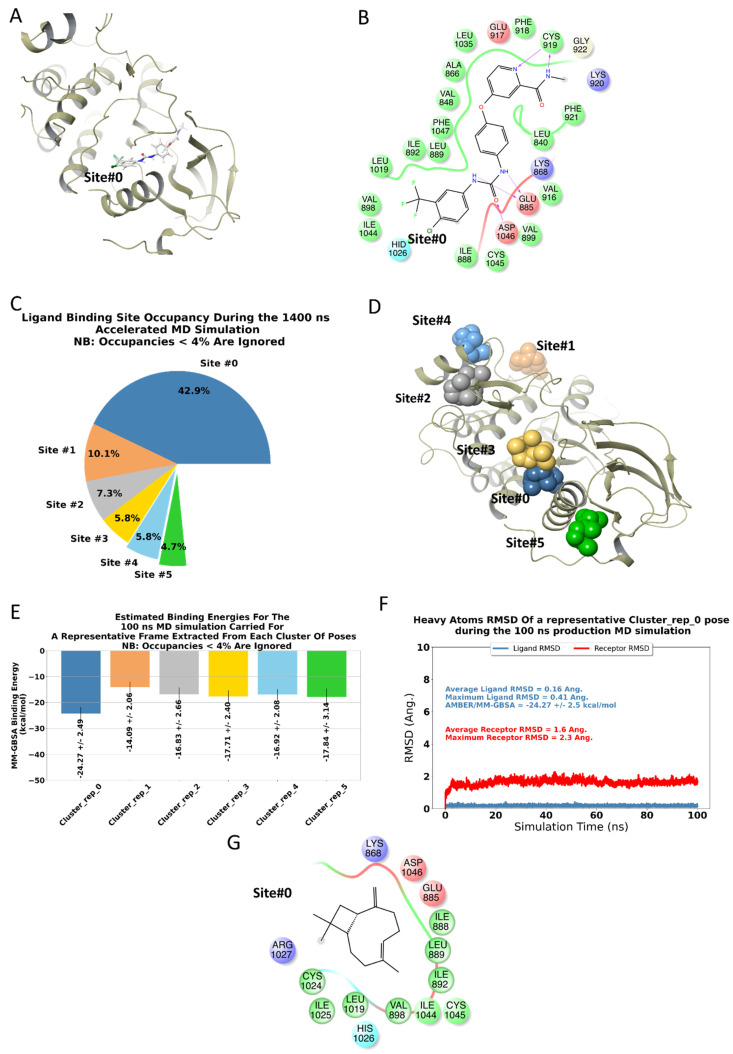
BCP has a binding site on the surface of VEGFR2 based on computational structural analysis: summary of the computational analyses performed in the current study. (**A**,**B**) represent the 3- & 2-dimensional ligand interaction diagrams, respectively, of the co-crystallized ligand, Sorafenib, with the kinase domain of VEGFR2 (PDB code: 3WZE). A pie chart representing potential BCP binding sites occupancies, obtained through clustering the full 1.4 μs accelerated MD trajectory (**C**). Representative conformations of BCP within these sites were given in figure (**D**) and color-coded using the same scheme displayed in the pie chart. Each of these representative conformations was subjected to 100 ns MD classical MD simulations, and the corresponding AMBER/MM-GBSA binding energy data was given in figure (**E**); again, with the same color codes of Figures (**C**,**D**). To enhance the clarity of the figures, we decided to depict the representative conformations and binding energies plots only for the top 6 clusters. Notably, Site#0 represents the most probable site of BCP, given the high fractional binding site occupancy and the better binding affinity compared to other sites. The RMSD plots presented in Figure (**F**) of the classical trajectory of BCP bound to Site#0 show stable trajectories for the ligand, and the receptor, a 2D ligand interaction diagram of BCP within Site#0 was given in Figure (**G**).

As BCP inhibited VEGF secretion from HUVECs, computational structural analysis was performed to understand the probable binding site(s) of BCP on VEGFR2. Our 1.4 μs accelerated MD trajectory suggested few potential sites on the surface of VEGFR2. For simplicity, we have denoted these sites according to the cluster identification number. To give a structural description of these sites relative to a traditional kinase inhibitor binding site, the 2D & 3D ligand interaction diagrams of sorafenib, a well-known competitive VEGFR2 inhibitor, bound to VEGFR2 are depicted in Figure 3A,B, where it can be seen that sorafenib formed two strong H-bonds with CYS919 through its N-methylpyridine-2-carboxamide group.

The ligand then extended to the lipophilic site (Site#0) through the 4-chloro-3-(trifluoromethyl)phenyl group [25]. This site was formed from highly lipophilic residues that include VAL898, LEU1019, ILE892, ILE1044, LEU889, and ILE888. As depicted in Figure 3C,D, clustering showed that BCP occupies Site#0 for ~42.9% of the total simulation time. Furthermore, as depicted in Figure 3E, BCP binding to Site#0 achieved the lowest AMBER/MM-GBSA binding energy in all the clustered poses (AMBER/MM-GBSA = −24.27 ± 2.49 kcal/mol). The bound pose of BCP at Site#0 achieved an overall stable MD trajectory (depicted in Figure 3F), with an average ligand RMSD of 0.16 Å and a maximum of 0.41 Å. The same trend was observed for the receptor RMSD that achieves an average of 1.6 Å and a maximum of 2.3 Å. Taken together, it seems that Site#0 at the surface of VEGFR2 is the most probable binding site for BCP. The 2D ligand interaction diagram of a sample BCP bound to Site#0 was given in Figure 3G. Although other potential sites are depicted in Figure 3, these sites, however, exhibited low binding affinities and reduced populations compared to Site#0. Of course, further structural and mutational analyses are required to confirm these findings. Being devoid of any heteroatoms, there is no chance of BCP to form stable H-bond interactions similar to what was formed by sorafenib or other traditional kinase inhibitors with hinge residues. Considering this fact, BCP can then be grouped with type-III kinase inhibitor [26]. In type-III inhibitors, the binding of the small molecule does not prevent the binding of ATP; however, the inhibitor will render the enzyme inactive even in the presence of a bound ATP molecule. The proposed mode of binding is similar to TAK-733 in its co-crystal with the human mitogen-activated protein kinase 1 (MEK 1, PDB code: 3PP1) [27].

### 2.5. BCP Blocked the Outgrowth of Microvessels of Rat Aorta

**Figure 4:**
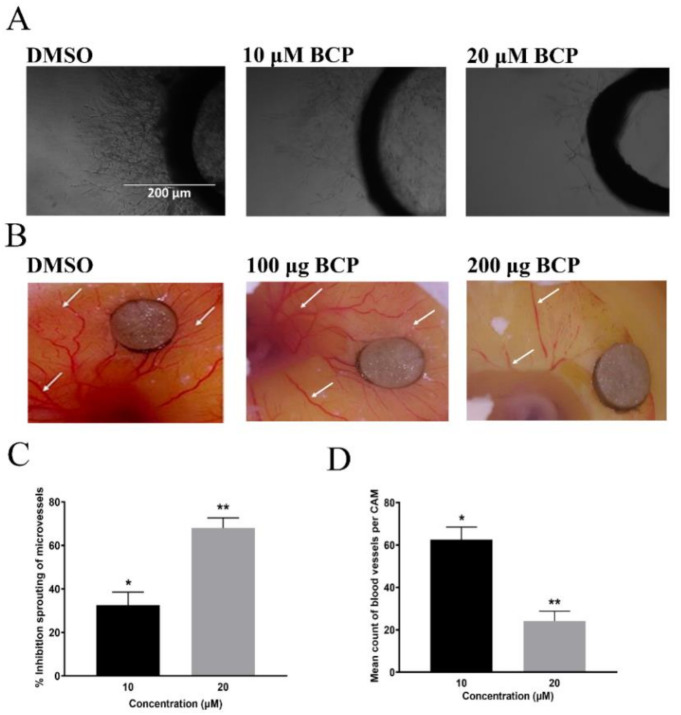
BCP inhibited angiogenesis ex vivo and in vivo. Photomicrographs showing the anti-angiogenic effect of BCP on the sprouting of microvessels in the rat aortic ring assay after treatment with (0.5% DMSO), 10, and 20 µM BCP. Representative images were taken on the 5th day of the experiment using an inverted phase-contrast microscope at 4× magnifications with the scale indicating 200 µm (**A**). Photomicrographs showing anti-angiogenic effect of BCP against chick embryonic CAM assay. Representative images of BCP on neovascularization in the chorioallantoic membrane, where the white arrows indicate the growth of new blood vessels indicating angiogenesis (**B**). Effect of BCP on rat aortic ring assay (**C**). Effect of BCP on the chick embryonic CAM assay (**D**). The data were represented as the mean ± SD (*n* = 3). * *p* < 0.05 and ** *p* < 0.01 compared to DMSO-treated control group.

The anti-angiogenic activity of BCP was investigated using an ex vivo model involving 3D rat aortic ring. Treatment of 1% DMSO showed a clear outgrowth of microvessels from the aortic rings, whereas, BCP treatment reduced the outgrowth of the aortic rings with IC_50_ of 15.6 ± 2.3 μM (Figure 4A). 

BCP revealed a significant dose-dependent anti-angiogenic effect on the rat aortic explants with 10 and 20 μM BCP reducing the vascularization by 32.06% ± 5.90 and 68.01% ± 3.08, respectively (Figure 4C). These data strongly suggest that BCP inhibited angiogenesis ex vivo.

### 2.6. BCP Blocked Angiogenesis in CAM Assay

The anti-angiogenic activity of BCP was further explored using an in-vivo chicken embryo chorioallantoic membrane (CAM) model. As illustrated in Figure 4B, the DMSO-treated CAM showed normal vasculature structure with major and minor vessels along with dendritic branching arrangement. Contrarily, vascularization architecture in chicken embryo was deformed by BCP with significant inhibitory percentages (*p* < 0.05) in comparison to the control (Figure 4D). Quantitative analysis revealed that the percentages of neovascularization inhibition by BCP in CAMs were 62.06 ± 5.09% and 24.15 ± 4.68% of 10 μM and 20 μM, respectively.

### 2.7. BCP Inhibited Tumor Growth in an HCT-116 Cells Transplanted Mouse Xenograft Model

**Figure 5:**
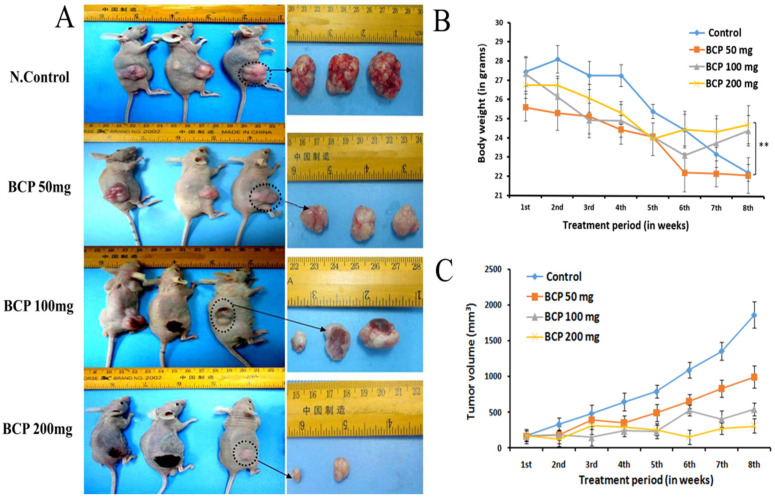
BCP inhibited tumor growth in an ectopic tumor model. Photographic images represent a subcutaneous xenograft mice model with regard to tumor growth and tumor sizes. Mice were injected subcutaneously with HCT 116 cells 8 days before treatment. Different doses of BCP were supplemented for 8 weeks and the tumor size was measured (**A**). The dose-response relationship of BCP on the body weight of vehicle-treated (control) and BCP-treated animals (**B**). ** *p* < 0.01 compared to control group. The dose-response relationship of BCP on tumor size of vehicle-treated (control) and BCP-treated animals. Each point represented the mean ± SD of 6 tumors (**C**).

The in-vivo anti-tumor effect of BCP was examined on an ectopic model, using NCr nu/nu nude mice, transplanted with colorectal cancer cell line, HCT 116. BCP was orally administered for a period of 8 weeks. Tumors from the mice in the control group grew rapidly, with an increase in the mean tumor volume from 333 mm^3^ to 647 mm^3^ within 2 weeks. The result exhibited a remarkable reduction in the tumor size in all BCP-treated animals in a dose-dependent manner (Figure 5C). 

**Figure 6:**
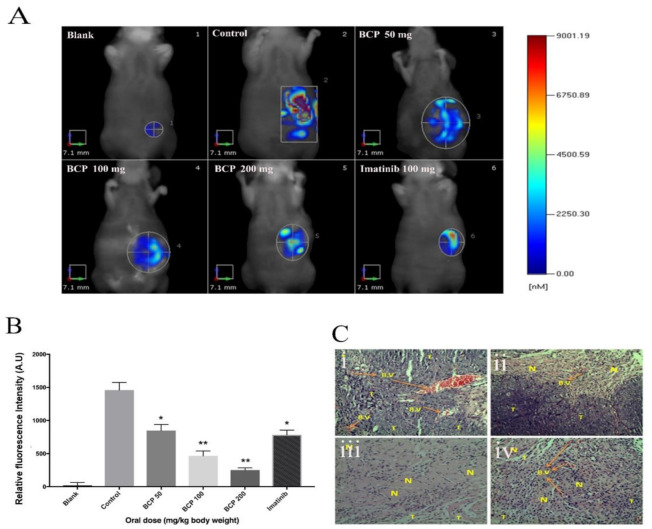
BCP inhibited tumor growth in an orthotopic tumor model as detected by Fluorescent Molecular Tomography. Photographic images represent the anti-tumor activity of BCP. HCT 116 tumor cells implanted orthotopically in the cecal wall of the nude mouse; (**A**) (1) Blank: normal mice, (2) negative control: untreated mice bearing tumor, (3) mice treated with 50 mg/kg of BCP, (4) mice treated with 100 mg/kg of BCP, (5) mice treated with 200 mg/kg of BCP and (6) positive control: mice treated with 100 mg/kg of Imatinib. (**B**) Graphical representation of the fluorescent intensity of the tumor in mice treated without and with BCP detected by Fluorescent Molecular Tomography. All data were represented as mean ± SD, *n* = 5, * *p* < 0.05 and ** *p* < 0.01 compared to control. (**C**) Cross-sections of orthotopic tumor tissues stained with hematoxylin/eosin. The pictures were captured at 20× magnification. (**i**) Negative control with more blood vessels, (**ii**) 100 mg/kg of BCP, (**iii**) 200 mg/kg of BCP, and (**iv**) positive control: 100 mg/kg of imatinib. (B.V) refers to the blood vessels, (T) refers to viable tumor cells, and (N) refers to necrotic/apoptotic tumor cells.

BCP (100 mg/kg) showed a significant anti-tumor activity with ∆T/∆C = 0.27% on 7th week post-cell inoculation day. Similarly, BCP at the dose of 200 mg/kg revealed remarkable tumor reduction (0.07%, *p* < 0.001). Additionally, the morphology of excised tumors revealed obvious inhibition on the density of blood vessels in the tumors of BCP-treated groups compared to the tumors in the control group (Figure 5A). Notably, the body weight of the 100 and 200 mg/kg BCP-treated animals was significantly higher than the control groups (Figure 5B). All the mice in the control group treated with water and Tween 80 died at 8th week of tumor inoculation, but BCP significantly prolonged the animals’ survival rate. Furthermore, an orthotopically implanted human colorectal cancer cells, HCT 116 into a xenograft nude mice model was also used to study the effects of BCP on tumor growth. As illustrated in Figure 6A, FMT quantification showed a fluorescent signal from the accumulation of labeled reagent in the tumor of colorectal cancer in the abdominal region. High intensity of fluorescence signal was detected in the mice treated with water and Tween 80 (control), whereas BCP-treated animals exhibited a dose-dependent inhibition of the fluorescence signal intensity. These data are comparable with the standard drug Imatinib (Figure 6B). Moreover, following the hematoxylin/eosin staining of the excised tumor tissue cross-sections, the morphology revealed a significant reduced tumor vascular density in the tumors from BCP-treated groups compared to the tumors from water and Tween 80-treated group (Figure 6C). These results correlated very well with the observations from the ectopic model.

### 2.8. Detection of Ultra-Structural Morphology of Apoptotic HCT 116 Cells by TEM

**Figure 7:**
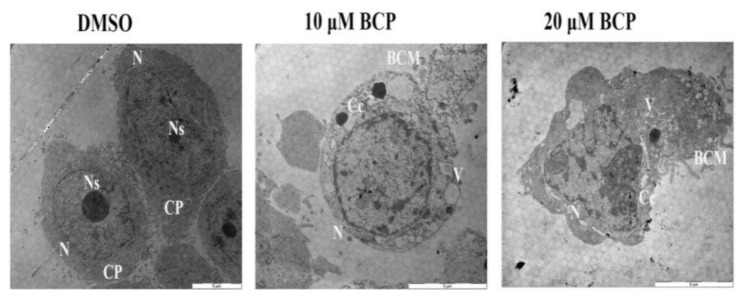
Ultrastructural micrographs using the TEM showing the apoptotic features of HCT 116 cells treated with BCP. HCT 116 cells treated with vehicle (0.5% DMSO) revealed intact cell membrane, normal cytoplasm (CP) with clear nucleus (N), and nucleolus (Ns). In contrast, BCP-treated cells revealed numerous cytoplasmic vacuoles (V), blebbing of the cell membrane (BCM), chromatin condensation (Cc), and disappearance of the nucleolus (Ns), suggesting the features of apoptotic cell death. The pictures were taken after 24 h treatment of vehicle or BCP at 1600× magnification.

HCT 116 cells showed noticeable ultra-structural morphological changes after treatment with 10 and 20 μM of BCP for 24 h, illustrating features of apoptotic events in these BCP-treated cells (Figure 7). Control HCT 116 cells supplemented with 0.5% DMSO showed complete cell membrane, normal cytoplasm with dense cellular contents (like nucleus and nucleolus), whereas cells supplemented with BCP (both 10 and 20 μM) demonstrated conspicuous signs of apoptosis including blebbing of the cell membrane, formation of various sized vacuoles, chromatin condensation and nucleolus shrinking, supporting apoptosis induction. The nucleus of the cells treated with 10 μM BCP confirmed some changes and contained one nucleolus, but it was smaller in size and lost the nucleolus at the higher concentration (20 μM). This confirms that the HCT 116 cells undergo cell death via apoptosis induction upon treatment with BCP.

### 2.9. Effect of BCP on the Expression of Apoptotic Proteins in HCT 116 Cells

**Figure 8:**
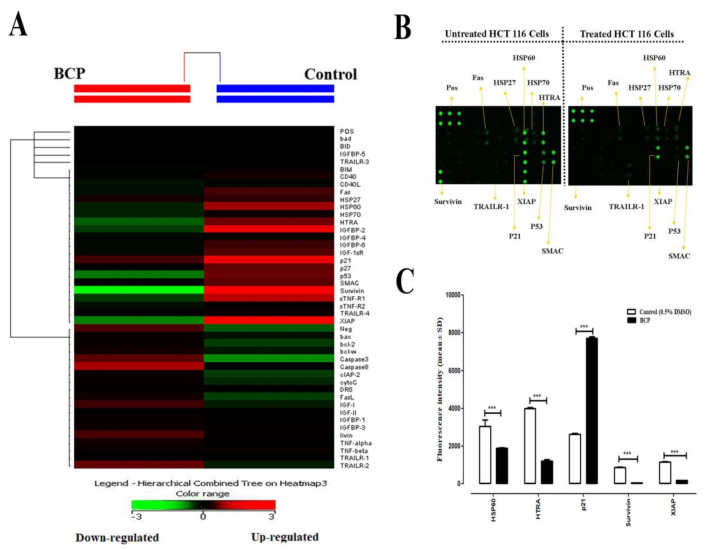
Effect of BCP on the expression of apoptosis-related proteins in HCT 116 cells. Cells were treated with 20 μM BCP for 24 h. Cells were lysed, the whole-cell protein was extracted, and protein array was performed. (**A**) A cluster diagram (heat map) shows signal intensities from each protein. (**B**) Images of the apoptotic markers were presented for the untreated and BCP-treated cells. Positive Control Spots (POS) were used for normalization and orientate the arrays. (**C**) Relative fold change in the expression levels of various pro- and anti-apoptotic proteins after treatment of BCP with HCT 116 cells. The statistical analysis was performed by assuming a value of 1 in control for reported proteins. The data was represented as the mean ± SD (*n* = 3). *** *p* < 0.001 compared to DMSO-treated control group.

Data from the human apoptosis antibody array kit are shown in Figure 8. The representative images of the array from untreated and BCP-treated cells (Figure 8A), the heat-map image showing the degree of expression of various apoptotic proteins detected by the protein array kit (Figure 8B) along with the quantification of levels of the different up- and down-regulated proteins (Figure 8C) were presented. Treatment of HCT 116 cells with BCP significantly downregulated the expression of major anti-apoptotic proteins like survivin (*p* < 0.001) and X-linked inhibitor of apoptosis protein (XIAP) (*p* < 0.001), along with the serine protease high-temperature requirement factor (HTRA) known to be involved in caspase-dependent apoptosis and a member of heat shock proteins family, HSP60 (*p* < 0.001) (Figure 8A). These markers are associated with oxidative stress in the cells. Among the 43 apoptosis-related proteins that could be detected by the selected kit, there were no other anti-apoptotic markers that were significantly up-regulated in their relative expression after treatment of BCP for 24 h as compared to control. The cell proliferation repressor protein, p21, was the only protein significantly up-regulated (*p* < 0.001) by BCP in HCT 116 cells (Figure 8C).

## 3. Discussion

Angiogenesis, resulting in the development of tumor vascularization, is an indispensable process for cancer invasion and metastasis. The angiogenic process implicates various steps, such as proliferation, differentiation, motility, and formation of tube-like structures by the endothelial cells, which integrate into the outer surface of sprouting microvessels. This whole process can be triggered by several growth stimulators. For instance, high expression of VEGF-A alone is sufficient to stimulate angiogenesis in a quiescent vasculature [28]. Accumulating reports have highlighted the importance of sustained normalization and inhibition of abnormal tumor vessels for combating metastasis [29]. In this study, the anti-tumor activity of BCP, an isolated compound from *Aquilaria crassna* [20], was evaluated on a series of anti-angiogenesis-related assays using both in vitro and in/ex vivo approaches along with a computational modeling study to understand the probable binding site of BCP with VEGFR. The result showed that BCP showed an anti-proliferative effect against the endothelial cells with an IC_50_ of 41.6 ± 3.2 μM. Moreover, BCP effectively blocked the migration and formation of endothelial cells’ network assembly at sub-toxic doses on HUVECs. The migration of endothelial cells is one of the critical steps in angiogenesis, initiating the ability to invade surrounding spaces. The results of the scratch migration assay revealed that BCP could obstruct endothelial cell motility at concentrations of 10 and 20 μM. Following the cell migration into the perivascular channels in the blood vessels, capillary tube assembly takes place to facilitate the formation of endothelial cells in a 3D structure, which is considered as a second hallmark of neovascularization [30]; BCP profoundly suppressed the formation of capillary-like structures in a concentration-dependent manner. Folkman suggested that every 100 tumor cells require at least one to ten vascular endothelial cell(s) to grow solid tumors [31]. VEGF is the predominant factor secreted by tumor cells that trigger endothelial cells to form new capillaries resulting in the progression of solid tumors. Moreover, HUVECs differentiation involves the activation of VEGFR-2, which mediates VEGF-induced endothelial cells differentiation into capillary-like tube structure networks. The possible binding of BCP to VEGFR might play a role with pronounced suppression of VEGF secretion by BCP, which consequently could have caused inhibition of proliferation and differentiation of HUVECs. The molecular dynamic simulation by computational structural analysis revealed Site#0 as the most plausible binding site of BCP at the surface of VEGFR2. The stable MD trajectory and ligand interaction analysis without forming any stable H-bond interactions led us to propose BCP to be grouped with type-III kinase inhibitors. As there are few other active binding sites observed in computational modeling, further structural and mutational analyses are required to clarify these findings. From the above results, it is evident that BCP interferes with the primary steps of angiogenesis process in endothelial cells, including cell migration and differentiation to the capillary-like structures, in addition to inhibition of a key promoter of angiogenesis, VEGF. The anti-angiogenic activity of BCP on microvessel outgrowth of ex vivo rat aortic ring assay exhibited a significant concentration-dependent relationship with IC_50_ of 15.6 ± 2.3 μM. Furthermore, the vascularization in chick embryos was also inhibited by BCP, indicating the anti-angiogenic activity of BCP in vivo.

Owing to the fact that most of the growth of solid tumors is closely linked to neovascularization, efficient blood supply is necessary to sustain tumor progression with oxygen and nutrients. Tumor angiogenesis is not only essential in the late stage of tumor development but is also crucial in the initiation step of tumor growth to clinically visible masses [32]. Therefore, blocking tumor angiogenesis has been widely accepted as a validated major approach in cancer prevention and promising cancer therapy [33]. In line with this rationale, we have tested the in vivo anti-tumor effect of BCP on nu/nu athymic nude mice using two animal models of tumors (ectopic and orthotopic), which physiologically mimics the microenvironment nature of human neoplasm. The result of the colorectal cancer ectopic model showed a remarkable reduction in the tumor size after 7–8 weeks of treatment with BCP-supplemented groups compared to the control group. Both morphological observation and microscopic biopsy of cross-sectional images revealed a noticeable reduction in the vascular density of tumors excised from BCP-treated animals indicating that BCP treatment causes a substantial reduction in the vascularization of tumors. The finding of the orthotopic mouse model clearly demonstrated that the treatment of the tumor-bearing mice with BCP at dose of 200 mg/kg and 100 mg/kg caused potent inhibition of colorectal cancer. Notably, a recent study also reported that 200 mg of BCP exerted anti-cancer and hypoglycemic activity in diabetic BALB/c mice grafted with CT26 colon cancer cell line. Interestingly, in that study, BCP suppressed arginine-specific mono-ADP-ribosyl transferase 1 (ART1) prompted glycolysis through the AKT/mTOR signaling pathway, which suggests that BCP can obstruct the proliferation and induce the programmed cell death of CRC cells [34].

During an apoptotic event, typical morphological alterations occur at the cellular and subcellular levels that can be studied using light inverted phase contrast and electron microscopic methods. It starts with cell shrinkage and weakening of interaction between cells followed by chromatin condensation and margination of the nuclear membrane, condensation of cytoplasm, tight packing of cellular organelles, degradation of cytoskeletal and nuclear proteins and cross-linking, membrane blebbing, fragmentation of cell into compact apoptotic bodies containing cytosol, condensed chromatin and cellular organelles with intact morphology and finally the engulfment of apoptotic bodies resulting in the removal of faulty/cancerous cells from affected areas [35]. The current study employed both TEM and protein array approaches to study the various apoptotic features induced in HCT 116 cells after exposure to BCP. In the protein apoptosis array study, only 5 of the 43 proteins showed substantial alteration when treated with 20 μM of BCP. These proteins included HSP60, HTRA, p21, survivin, and XIAP. Exposure of HCT 116 cells to BCP resulted in significant down-regulation in the expression of two major anti-apoptotic markers, including XIAP and survivin. These proteins are known to arrest apoptosis by direct inhibition of caspases-9 and -3/7 [36]. Moreover, high expression of survivin has been universally associated with resistance to apoptosis, higher tumor grade, increased metastasis, and resistance to therapy in virtually every human tumor [37]. Our finding of survivin downregulation is in accordance with the previous report by Park et al., wherein survivin expression was remarkably decreased in both breast and prostate cancer cells after treatment with BCP oxide [38]. Apart from survivin and XIAP, BCP exposure also decreased the expression of the heat shock protein, HSP60, which has been reported to inhibit activation of mitochondria-mediated apoptosis through halting the release of cytochrome C and up-regulation of survivin in colon cancer [39]. On the other hand, p21 is the only overexpressed protein by BCP in HCT 116 cells. Despite the dual role of p21 (anti-apoptotic/pro-apoptotic), various human solid malignant tumors such as colorectal, lung, breast, head, and neck cancers are significantly associated with reduced p21 expression [40]. Unlike p53, p21 may not be a traditional tumor suppressor protein, but it synergizes with tumor suppressors and antagonizes oncogenes to protect against cancer [41]. In our study, the remarkable increase in the expression of p21 suggests that overexpression of p21 in HCT 116 cells may mediate suppression of cellular proliferation and trigger apoptosis. However, HTRA is a pro-apoptotic marker that is released upon the induction of the mitochondrial pathway. In this study, HTRA was down regulated by BCP. Nonetheless, this alteration did not prevent the activation of apoptosis by BCP in CRC cells. We have previously reported the inhibitory effect of BCP on the proliferation of 9 different cell lines (seven tumor and two normal cell lines of different tissue origin including colorectal, pancreas, breast, prostate and leukemia) [24]. Among these cell lines, BCP showed the highest selectivity index (SI) towards the colorectal cancer cells, viz., HCT 116 with SI = 27.9, followed by HT 29 cells with SI = 19.6. Moreover, HCT 116 is the cell line that was widely used to study tumorigenesis and metastasis of colorectal cancer in animal models. Therefore, we have used only HCT 116 cells in the current study. Further investigations involving different colorectal cancer cell lines are warranted in the future to validate the current findings and understand the underlying signaling mechanism(s) involved in the anti-colorectal cancer effect of BCP.

## 4. Materials and Methods

### 4.1. Materials

Human Apoptosis Antibody Array C1 and human VEGF-A ELISA kits were purchased from RayBiotech, Inc., (Peachtree Corners, GA, USA). Human colorectal carcinoma cells (HCT 116) and human umbilical vein endothelial cells (HUVECs) were purchased from ATCC (American Type Culture Collection, Rockville, MD, USA). The cell culture media M199 and RPMI 1640, cell culture-grade dimethyl sulfoxide (DMSO), and all other reagents were obtained from Sigma-Aldrich (Darmstadt, Germany). BCP was dissolved in DMSO and 5% Tween 80 in distilled water (*v/v*) for in vitro and in vivo studies, respectively.

### 4.2. Cell Culture

HCT 116 and HUVEC cells were preserved in the cell culture laboratory, School of Pharmacy, USM, and maintained in modified RPMI medium and M199 containing 10% fetal bovine serum (FBS), respectively. Cells were grown in an incubator maintained at 37 °C in a humidified 5% CO_2_ conditions.

### 4.3. Cell Proliferation Assay

3-(4,5-dimethyl-2-thiazolyl)-2,5-diphenyl-2*H*-tetrazolium bromide (MTT) test was utilized to measure the proliferation of HUVECs. The cells (1.5 × 10^4^) were suspended in 100 μL of fresh culture medium and seeded in each well of a 96-well plate followed by overnight incubation at 37 °C in 5% CO_2_. Then, cells having 80% confluence were treated with different concentrations of BCP (6.12–100 µM) with DMSO (0.5%) as the negative (solvent) control. After 48-h incubation, following the aspiration of the medium, 5 mg/mL of MTT solution (prepared in sterile PBS) was added, and the plate was incubated again for 4 h. Afterwards, following the addition of 200 μL DMSO per well, the absorbance was measured at 570 nm using infinite^®^Pro200 TECAN (Männedorf, Switzerland) with 620 nm as the reference wavelength.

### 4.4. Migration Assay

Migration assay was performed to assess the effect of BCP on the chemotactic capability of HUVECs. Briefly, HUVECs were seeded in a 6-well plate and incubated to form a confluent monolayer. Then, a wound was created in each well with a 200 µL micropipette tip. Subsequently, the detached cells were removed by washing the wells twice with PBS, and then treated with DMSO (0.5%) or BCP at various concentrations. After 24 h, photographs of the wounds were captured through a digital camera followed by the measurement of the width of wound-free cells under an inverted light microscope (Leica Quin; Microsystems computerized imaging system, USA). A minimum of 30 readings per field and 10 fields per well were captured. By comparing with control, the mean percentage of migration inhibition was calculated by using the following formula:

% migration inhibition = [1 − (Ds/Dc)] × 100

where Ds = distance traveled by the cells with treatment of test agent (BCP) and Dc = distance traveled by the cells with treatment of vehicle (DMSO).

### 4.5. Tube Formation Assay

A well-established reported method (endothelial cell tube formation plate system for angiogenesis) was used to analyze the capillary formation ability of HUVECs on Matrigel^®^ matrix [42]. Briefly, the matrix was set to polymerization by incubating at 37 °C and 5% CO_2_ for 45 min. Then, HUVECs were harvested by trypsinization and seeded in a six-well plate at seeding density of 2 × 10^5^ cells/well/100 μL of extracellular matrix containing different concentrations of BCP. The tubular networks were analyzed after 6 h of incubation with photographs taken through a digital camera (DC300, Leica Microsystems Inc. Bannockburn, IL, USA) attached with an inverted light microscope (4× magnification). Measurement of the area covered by the tubular structures using the Scion image analysis program indicated quantitatively the inhibition of tube formation. The mean % of inhibition was calculated by following formula and data are expressed as mean ± SD:

% of inhibition of tube formation = (1 − (Area T/Area C)) × 100

where Area T: the area of tubules in the BCP-treated wells and Area C: the area of tubules in the control (untreated) wells.

### 4.6. Measurement of VEGF Expression in the Lysates of HUVECs

The concentration of VEGF in the lysates of HUVECs was measured using a Human VEGF-A ELISA kit (RayBio, Peachtree Corners, GA, USA) following the manufacturer’s protocol. Initially, HUVECs were treated with BCP (10 and 20 μM) or 0.5% DMSO (as solvent control) for 6 h. Wizard^®^ SV Lysis buffer (Promega, Madison, WI, USA) was used for preparation of cell lysates. The standards and the lysates of HUVECs were then pipetted into the wells (in triplicate) of the 96-well plate of the kit, which were coated with human VEGF specific antibody. The plate was incubated at 15 °C for 24 h to facilitate the efficient VEGF binding with the immobilized antibody. Then, following the washing of the cells with a wash buffer solution, anti-human VEGF biotinylated antibody was added to each well. The wells were subsequently washed followed by addition of HRP-conjugated streptavidin. Afterwards, addition of tetramethylbenzidine substrate solution resulted in blue color formation, with the intensity of color being proportional to VEGF content. Then, sulphuric acid (stop solution) was added to the wells, turning blue color to yellow. Lastly, the absorbance was measured using a microplate reader at 450 nm. A regression equation was used for the estimation of VEGF concentration (pg/mL of cell lysates) and the results were presented as the mean ± SD.

### 4.7. Computational Methods

The probability of BCP to bind on the surface of the VEGF receptor 2 (VEGFR2) was studied by computational structural analysis. For a comprehensive description of the conducted computational analyses, please consult the supporting materials attached to the manuscript (Supplementary Method S1). In brief, we saved 14 different docked poses of BCP bound to a model VEGFR2 kinase, PDB ID: 3WZE [25]. This was followed by classical and 1.4 μs accelerated MD simulations and binding energy calculations to investigate potential binding sites. For more details about the scheme and the selected parameters of the accelerated simulations, readers are encouraged to consult our recent study for applying accelerated MD (aMD) to the programmed cell death ligand protein, PD-L1 [43].

### 4.8. Ex Vivo Angiogenesis Model

#### 4.8.1. Experimental Animals

Sprague Dawley male rats (11–13 weeks old) were purchased from the animal house facility of the School of Pharmaceutical Sciences, USM, and kept there for one week to adapt with environment. Animals were supplied with sufficient food/water continuously in whole duration and placed in a clean ventilated environment. In-vivo experimentation was preformed according to the Animal Ethics Committee of USM and approved with the reference number (2014/(94) (673)).

#### 4.8.2. Preparation of Rat Aortic Segments into Culturing Plates

The rats were sacrificed by cervical dislocation following euthanization in CO_2_ chamber. A small mid-line incision was created around the abdominal and thoracic cavities at the sternum partition. Then, the aorta was cleaned quickly from fibro-fat tissues or blood clumps with special care to avoid injury and 1 mm length of aorta rings were cross-sectioned. Two layers (solidified and liquid) of culture medium were used for the preparation of tissue culture plates. A sterile 48-well microtitration plate having 300 μL of serum-free M199 medium (3 mg/mL fibrinogen and 5 mg/mL aprotinin) was used for this assay. Fresh rings were implanted followed by addition of 10 μL of thrombin (50 NIH U/mL) in each well. DMSO (negative control) and BCP (10 and 20 µM) were pipetted into the second layer of M199 medium. The plates were incubated for 24 h at 37 °C. On 5th day, photographs of the aortic rings were captured with highly magnified camera (4×). The length of outgrowth of the blood vessels from the primary tissue ex-plants was measured using the Leica Quin software package as an index of the angiogenic response. The percentage inhibition of angiogenesis was calculated by the following formula:

Percentage of neovascularization inhibition = (1 − (DS/DN.C)) × 100

where DS = the distance of blood vessels growth of BCP-treated well and DN.C = distance of blood vessels growth of DMSO-treated well.

The results are presented as mean percent inhibition of angiogenesis ± SD as compared to the negative control.

### 4.9. In Vivo Anti-Angiogenic Study by CAM Assay

The anti-angiogenic effect of BCP was investigated in vivo by utilizing a modified chicken embryo chorioallantoic membrane (CAM) assay technique, as reported earlier [44]. Ten fertile eggs were obtained from a local hatchery and incubated for five days in a humidified incubator at 37 °C. On day five, all the eggs were cleaned, sprayed with 70% ethanol. The eggs were kept in a horizontal position to allow the embryos to settle down; to prevent contact of CAM membrane with the upper eggshell. Consequently, a small hole was created softly on the upper surface of the eggshell with enough care to prevent CAM architecture. BCP was prepared in ethanol at 100 and 200 mg/mL and the clean filter paper discs (5 × 5 mm) soaked with either DMSO (control) or BCP were placed on the CAM. Later on, the discs were kept for 20–30 min at 25 °C to be solidified. After that, the discs were implanted (1 disc/egg) onto the top of CAM at the opened window with extreme care at an area of fine blood capillaries. The window was covered with the eggshell and then by adhesive tape to prevent dehydration. The eggs were placed again in the incubator for a further 48 h. Finally, the egg window was removed again, and the disc area of CAM was imaged using a camera fitted on a dissecting microscope (SMZ-168, Motic, Wetzlar, Germany). The results were presented as the average of the percentage of inhibition ± SD of three experiments by using the following formula:

% of inhibition = (1−(S/N.C)) × 100

where S: number of blood vessels in eggs treated with BCP and N.C: number of blood vessels in eggs treated with DMSO (control).

### 4.10. In Vivo Anti-Tumor Studies

#### 4.10.1. Experimental Animals

Athymic NCr nu/nu nude mice were obtained from Eman Biodiscoveries (USM-Malaysia). Eight mice of same gender were put together in clean cages fitted with high-efficiency particulate air filters. Animals were constantly provided with sterilized food and water, and the autoclaved bedding was changed every 3 days. The protocol was approved by the Animal Ethics Committee at the USM (reference number 2014/(94) (672)).

#### 4.10.2. Establishment of Ectopic Model

The HCT 116 cell line was selected to establish the in vivo model of colorectal cancer. HCT 116 cells maintained in RPMI 1640 medium were collected by centrifugation at 1000 rpm for five min. The cell pellet was suspended in 200 µL of RPMI culture medium and kept on ice. The mice with an average weight of 25 g and aged from five to eight weeks were injected subcutaneously (8 mice/group) in the right flank with 5 × 10^6^ cells in 200 µL culture medium using 1 mL sterile syringe attached to 25 gauge needle. The injection site was inspected for 30 s with a sterile cotton swab to avoid any leakage of cells before the animals were returned to the cages. After the first week, when average tumor volume reached about 100 mm^3^, mice were divided into four groups of 6–8 animals each. The first group (control) was orally administrated with 200 μL of distilled water with Tween 80. The second, third and fourth groups were treated with 200, 100 and 50 mg/kg body weight of BCP, respectively. Changes in tumor size and body weight were measured following previously described equation [45]. The percentage of tumor reduction (anti-tumor efficiency) was calculated according to the Division of Cancer Treatment, NCI, NIH [46].

### 4.11. Orthotopic Mice Model

#### 4.11.1. Establishment of the Xenograft Tumor Model

The animals were anesthetized using sodium pentobarbital (60 mg/kg). A 3–5 mm incision was made in the abdomen using a sterilized surgical scissor. HCT 116 cells (5 × 10^6^ in 200 µL RPMI media) were implanted surgically into the cecal wall of nude mice, five to eight weeks old and 20–25 g, using 25 G sterile needles. The injection site was inspected to ensure no leakage of cells. The incised nick was closed with a single suture. Antibiotics (penicillin/streptomycin) were applied to the sutured part with a sterile cotton swab.

#### 4.11.2. Treatment and Tumor Size Measurement

After 10–12 days of cell inoculation and complete wound healing, mice were grouped into five sets of 6–8 animals each. The treatment with BCP started on the 12th day after cell inoculation, where the first group was administered with 200 μL of distilled water with Tween 80 as a vehicle for control. The second, third and fourth groups were administered with 200, 100, and 50 mg/kg body weight of BCP, respectively. Group five received imatinib (as mesylate) at 100 mg/kg/day. Animals were supplemented once daily with their respective treatment regimens by oral gavages for five weeks. The tumor size was measured using fluorescent molecular tomography (FMT).

### 4.12. Demonstration of Apoptotic Characteristics of HCT 116 Cells by Transmission Electron Microscope (TEM)

The effect of BCP on cellular morphological alterations of HCT 116 was assessed using TEM following a previously described method [47]. HCT 116 cells were cultured in 25-mL culture flasks at a density of 1 × 10^5^ cells/mL in RPMI culture medium and incubated for 24 h at 37 °C supplied by 5% CO_2_. Later on, the medium was replenished by fresh RPMI medium (5 mL/flask) containing BCP samples at concentrations of 10 and 20 μM, and DMSO (0.5%) as a negative control. The flasks were re-incubated for further 24 h followed by harvesting of the cells by trypsinization and centrifugation to remove all content of the medium. Then, the cells were fixed using McDowell-Trump fixative solution and supplied with 1% osmium tetroxide. The cells were solidified in a 2% agar solution (Fisher Scientific, Toronto, ON, Canada), incised into small sections, and embedding in resin (1:1 of acetone: Spurr’s resin mixture) for 30 min and repeatedly every day for 5 days at 60 °C. Consequently, the specimens were embedded in resin and sliced precisely into semi-thin and ultrathin sections (1 and 0.1 μm thickness, respectively) using an ultramicrotome (Sorvall Ultramicrotome MT5000, DuPont, Liverpool, NY, USA). The semi-thin sections were prepared for staining using toluidine blue and to ensure the existence of the cells in the specimens. The ultrathin sections were collected in copper grids and stained with uranyl acetate and lead citrate. At the end of the experiment, cells were examined and photographed using a transmission electron microscope (TEM) (LIBRA 120 EFTEM, Carl-Zeiss, Oberkochen, Germany) at 1600× magnification. The ultra-alterations of the BCP-treated cells were compared with DMSO-treated cells.

### 4.13. Human Apoptosis Antibody Array

The apoptosis-related proteome profile C1 Kit (RayBio^®^ Human Apoptosis Antibody Array C1 Kit, cat# AAH-APO-1-2, RayBio, Peachtree Corners, GA, USA) was used to investigate the effects of BCP on the expression patterns of 43 pro-apoptotic and anti-apoptotic markers in HCT 116 cells. Two sets of HCT 116 cells (1 × 10^5^ cells/mL) were cultured in T25 flasks, and supplemented with 0.5% DMSO and 20 μM BCP for 24 h for control and treated group, respectively. Subsequently, the cells were lysed in 1× lysis buffer and protease inhibitors provided in the kit. The assay was performed using the 8-well glass plate following the manufacturer’s protocol. Finally, an imaging system (Gel-Pro Imager, Media Cybernetics, Inc., Silver Spring, MD, USA) was used to measure the intensities of each array dot and normalized against the internal control.

### 4.14. Statistical Analysis

Data are expressed as mean ± standard deviation (SD), where the statistical analysis was carried out using GraphPad Prism version 6 (GraphPad Software, La Jolla, CA, USA). Quantitative data were analyzed by one-way ANOVA for all the experiments, whereas two-way ANOVA was used to analyze the apoptosis antibody array result. The difference was considered to be significant at *p* < 0.05.

## 5. Conclusions

The current study demonstrated that BCP suppressed angiogenesis by inhibiting vital functions of endothelial cells, such as proliferation, migration, and synthesis of VEGF in the endothelial cells, possibly by blocking the putative Site#0 on VEGFR2. Furthermore, BCP showed remarkable anti-tumorigenic activity in human colorectal tumor xenograft models (both ectopic and orthotopic). In addition, BCP induced apoptosis through downregulation of the expression of HSP60, HTRA, 19urviving, and XIAP along with the upregulation of p21. Taken together, these results suggest that BCP can serve as a promising anticancer agent for CRC therapy.

## Data Availability

Not applicable.

## Supplementary Materials

The following are available online at https://www.mdpi.com/article/10.3390/ijms221910550/s1 for Method S1.
