# Supplementary material for: β-Caryophyllene Induces Apoptosis and Inhibits Angiogenesis in Colorectal Cancer Models
Source: Int J Mol Sci. 2021 Sep 29;22(19):10550. doi: 10.3390/ijms221910550 (PMC8508804; doi:10.3390/ijms221910550)
Supplement: Supplementary file 1 [file ijms-22-10550-s001.zip › ijms-1353313-supplementary.pdf]

Supplementary S1

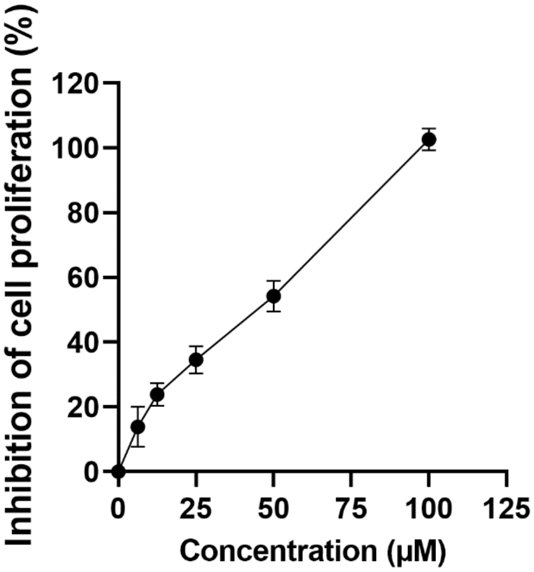

BCP inhibited proliferation of HUVECs *in vitro*. The concentration-dependent effect of BCP on the proliferation of HUVECs for 24 h was assessed by MTT assay. Data were represented as mean ± SD (n=3).
